# Development of a Curative Therapeutic Vaccine (TheraVac) for the Treatment of Large Established Tumors

**DOI:** 10.1038/s41598-017-14655-8

**Published:** 2017-10-27

**Authors:** Yingjie Nie, De Yang, Anna Trivett, Zhen Han, Haiyun Xin, Xin Chen, Joost J. Oppenheim

**Affiliations:** 1 Cancer and Inflammation Program, Center for Cancer Research, National Cancer Institute, Frederick National Laboratory for Cancer Research (FNLCR), Frederick, Maryland USA; 2 Guizhou Provincial Peoples’ Hospital, Guiyang, Guizhou Province China; 3 University of Macau, Macau, China

## Abstract

Harnessing immune system to treat cancer requires simultaneous generation of tumor-specific CTLs and curtailment of tumor immunosuppressive environment. Here, we developed an immunotherapeutic regimen capable of eliminating large established mouse tumors using HMGN1, a DC-activating TLR4 agonist capable of inducing anti-tumor immunity. Intratumoral delivery of HMGN1 with low dose of Cytoxan cured mice bearing small (∅ ≈ 0.5 cm), but not large (∅ ≈ 1.0 cm) CT26 tumors. Screening for activators capable of synergizing with HMGN1 in activating DC identified R848. Intratumoral delivery of HMGN1 and R848 plus Cytoxan eradicated large established CT26 tumors. The resultant tumor-free mice were resistant to subsequent challenge with CT26, indicating the generation of CT26-specific protective immunity. This immunotherapeutic regimen caused homing of tumor-infiltrating DC to draining lymph nodes and increased infiltration of T cells into tumor tissues. Cytoxan in this regimen could be replaced by anti-CTLA4) or anti-PD-L1. Importantly, this immunotherapeutic regimen was also curative for large established mouse Renca and EG7 tumors. Thus, we have developed a curative therapeutic vaccination regimen dubbed ‘TheraVac’ consisting of HMGN1 and R848 plus a checkpoint inhibitor, that can, without using exogenous tumor-associated antigen(s), eliminate various large tumors and induce tumor-specific immunity.

## Introduction

Cancer immunotherapies that harness host immune system to kill cancer cells have gained considerable ground over the past decade as witnessed by the clinical successes of adoptive transfer of either tumor-infiltrating lymphocytes (TILs) or T cells with chimeric antigen receptors, as well as therapeutic antibodies against either tumor cells (e.g. Rituximab, Trastuzumab, etc) or suppressive immune-checkpoint molecules (e.g. anti-CTLA4, anti-PD-1/PD-L1)^1–5^. Active immunotherapies such as vaccinating cancer patients with various forms of TAAs or *ex vivo* activated autologous DCs have so far, however, not led to robust and durable antitumor immune responses capable of eliminating preexisting tumors or markedly prolonging patients’ survival^3,4,6^. There are many obstacles to therapeutic tumor vaccine approaches. It is costly and difficult to determine the precise TAAs for an individual cancer patient since cancer of the same type at different stages may express different dominant TAAs due to continued mutations^7^. Even if the dominant TAAs are known for some cancer types, it remains difficult to induce sufficient number of antitumor CD8 effector cells^6,8^. Furthermore, the potent immunosuppressive microenvironment in cancer tissues often nullify antitumor immunological effector mechanisms^4,6,8^. Therefore, novel therapeutic antitumor approach(es) should be developed that can, without the need for identifying dominant TAAs, induce/amplify protective antitumor immune responses and overcome some of the immunosuppressive components, such as immunosuppressive cells [e.g. regulatory T cells (Treg), myeloid-derived suppressor cells, tumor-associated macrophages (TAM), etc], inhibitory immune checkpoints [e.g. CTLA4, PD-1/PD-L1, lymphocyte activation gene-3, indoleamine 2,3-dioxygenase, etc.], and/or immunosuppressive mediators, [e.g. interleukin 10 (IL-10), transforming growth factor β, vascular endothelial cell growth factor, etc.]^6,8^.

Tumor-infiltrating DCs (tDCs) have an immature and/or paralyzed phenotype because they tend to express low costimulatory molecules, high levels of inhibitory molecules and receptors, and impaired antigen-present capacity^9–11^. tDCs also contribute to the immunosuppressive microenvironment in tumor tissues by generating inhibitory signals and inducing Tregs^8,10,12–14^. Induction of sufficient maturation/activation of tDCs is likely to ameliorate the immunosuppressive tumor microenvironment and trigger antitumor immune response, and therefore is essential for the success of immunotherapy against tumors. Removal/blockade of the immunosuppressive components in cancer tissues can also be achieved using depleting antibodies specific for immunosuppressive cells, neutralizing antibodies against the inhibitory immune checkpoint molecules and mediators, or reagents capable of inhibiting the generation and/or function of inhibitory components^1,2,6,8,15–18^. In addition, antitumor immunity can be enhanced by the use of various adjuvants such as damage-associated molecular patterns, pathogen-associated molecular patterns, and certain delivery systems (e.g. virosome, immunostimulating complexes, QS-21, AS-04, etc) capable of promoting antigen uptake by DCs^6,8,19–23^.

Alarmins are a subset of damage-associated molecular patterns capable of inducing both the recruitment and activation of DCs that can promote specific immune responses against various antigens including many TAAs^19,23–27^, and therefore are adjuvants with the potential of augmenting the induction of antitumor immunity. A recently identified alarmin, HMGN1, is particularly attractive in this respect, because it is a TLR4 ligand that preferentially promotes Th1 immune response^27^. Hmgn1^−/−^ mice are more susceptible to carcinogenesis and have lower resistance to implanted tumors^19,28^, and HMGN1 has been shown to be critical for the generation of antitumor immunity against mouse thymoma and melanoma^19^. We therefore investigated whether HMGN1 could be used in the development of an immunotherapeutic regimen capable of eliciting robust antitumor immune responses and treating large established tumors.

## Results

To determine its antitumor effect, recombinant HMGN1 was injected intratumorally (i.t.) into Balb/c mice bearing CT26 colon tumors of approximately 0.5 cm in diameter. The i.t. route was chosen to promote the activation of APCs infiltrating tumor tissues by HMGN1, particularly tDCs. We hypothesized that activation of tDCs that are already loaded with tumor-associated antigen(s) (TAAs) would promote the induction of CT26-specific immune responses that, in turn, mediates elimination of tumor cells. HMGN1 at 1~25 μg/mouse dose-dependently inhibited, but did not halt, the growth of CT26 tumors (sFig. 1). The failure of HMGN1 by itself to halt the growth of CT26 tumors might be due to the immunosuppressive tumor microenvironment that hindered the elimination of tumor cells by CT26-specific cytotoxic T lymphocytes (CTLs). We therefore included Cytoxan (CY) to the treatment protocol since CY at low doses acts to suppress Tregs in both mice and humans^18,29,30^. Balb/c mice bearing CT26 tumors of 0.5~0.7 cm in diameter were treated with one intraperitoneal (i.p.) dose of CY at 2 mg/mouse and four i.t. injections of HMGN1 (10 μg/mouse, twice weekly) alone or in combination. Although HMGN1 or CY alone impeded the growth of CT26 tumors, the combination of HMGN1 and CY treatment halted tumor growth and caused shrinkage and complete elimination of the small CT26 colon tumors (sFig. 2). Unfortunately, treatment of Balb/c mice harboring large (∅ = 0.9~1.1 cm) CT26 tumors with the combination of HMGN1 and CY only slowed down, but did not halt, the growth of larger CT26 tumors (data not shown).

**Figure 1:**
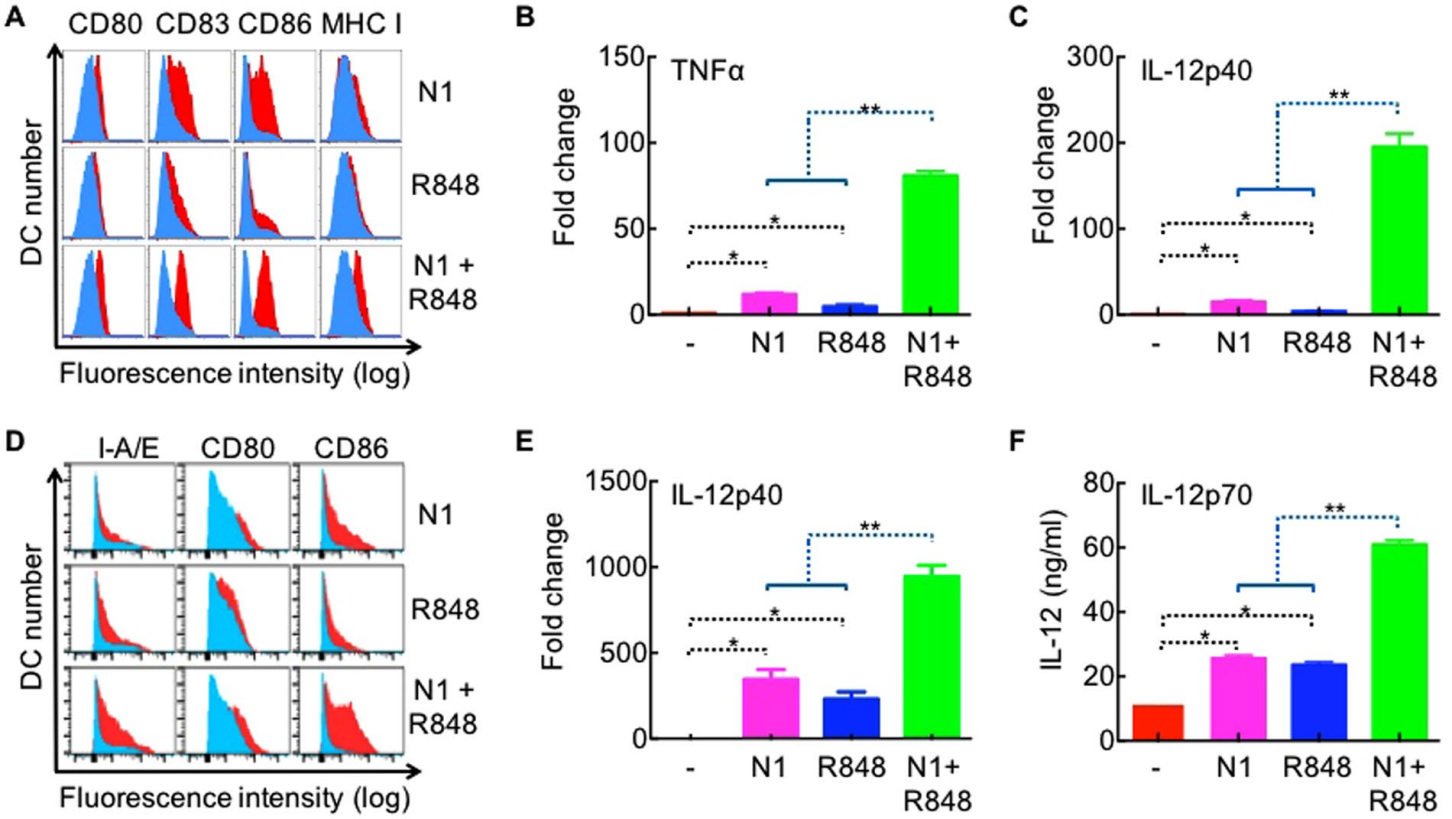
Synergistic activation of DCs by HMGN1 and R848. (**A**) Human monocyte-derived DCs treated at 0.5 × 10^6^/ml in RPMI 1640 medium in the absence or presence of HMGN1 (250 ng/ml) and/or R848 (20 ng/ml) for 48 h were analyzed by flow cytometry after immunostaining. Shown is the he expression of surface markers (CD80, CD83, CD86, and HLA-DR) as overlay histograms (blue = sham-treated, red = treated) of one experiment representative of three. (**B** and **C**) Human monocyte-derived DCs treated at 0.5 × 10^6^/ml in the absence or presence of HMGN1 (250 ng/ml) and/or R848 (20 ng/ml) for 6 h were used for RNA isolation and measurement of TNFα (**B**) and IL-12p40 (**C**) mRNA expression by qPCR. Shown are the average (mean + SD) of three separate experiments. *p < 0.05. **p < 0.05 when comparing the sum of HMGN1 and R848 groups with the group treated with a combination of HMGN1 + R848. (**D)** Balb/c BM-derived DCs were treated with HMGN1 (N1, 0.5 μg/ml) and/or R848 (0.1 μg/ml) for 48 h in humidified air containing 5% CO_2_, and subsequently stained and analyzed by flow cytometry (detailed in the Materials and Methods). Shown are the overlay histograms of surface I-A/E, CD80, and CD86 in response to indicated treatment (in red), with sham-treated DCs in light blue. (**E**) Balb/c BM-derived DCs were treated with HMGN1 (N1, 5 μg/ml) and/or R848 (20 ng/ml) for 6 h in humidified air containing 5% CO_2_, and IL-12p40 mRNA levels were quantitated by qPCR. Shown is the average (mean ± SD) of three experiments. DC treated with HMGN1 + R848 had higher IL-12p40 mRNA than the sum of R848-treated and N1-treated DC (**p < 0.001). (**F**) Balb/c BM-derived DCs were treated with HMGN1 (N1, 5 μg/ml) and/or R848 (20 ng/ml) for 24 h in humidified air containing 5% CO_2_, and IL-12p70 levels in the culture supernatants were quantitated by ELISA. Shown is the average (mean ± SD) of two experiments. DC treated with R848 + HMGN1 produced more IL-12p70 than the sum of R848-treated and N1-treated DC (*p < 0.05).

Since large mouse tumors (∅ ≥ 1 cm) are more likely to resemble the tumor load of human cancers at the time of diagnosis, it is essential to find a way to eradicate large mouse tumors. tDCs in larger tumors are likely to be more highly paralyzed^10,14,31^ and need additional immunoactivating signal(s) to be fully activated. We therefore screened a number of DC activators for those that could synergize with HMGN1 in the activation of human DCs, in particular, the induction of IL-12 (data not shown). This identified R848/resiquimod, a synthetic agonist for TLR7/8, which together with HMGN1 upregulated surface expression of costimulatory (CD80, CD83, and CD86) and MHC class I (HLA-A,B,C) molecules on human monocyte-derived and synergistically promoted DC expression of TNFα and IL-12p40 (Fig. 1A–C). Furthermore, when treated with HMGN1 or R848, mouse bone marrow-derived DCs also upregulated costimulatory (CD80 and CD86) and MHC class II (I-A/E) molecules on their surface (Fig. 1D) and IL-12 expression at both the mRNA (Fig. 1E) and protein (Fig. 1F) levels. Importantly, human and mouse DC treated with a combination of HMGN1 and R848 showed higher IL-12 expression than the sum of DCs treated with HMGN1 and R848 alone (Fig. 1C,E & F). Thus, HMGN1 and R848 synergistically promoted the activation of both human and mouse DCs.

**Figure 2:**
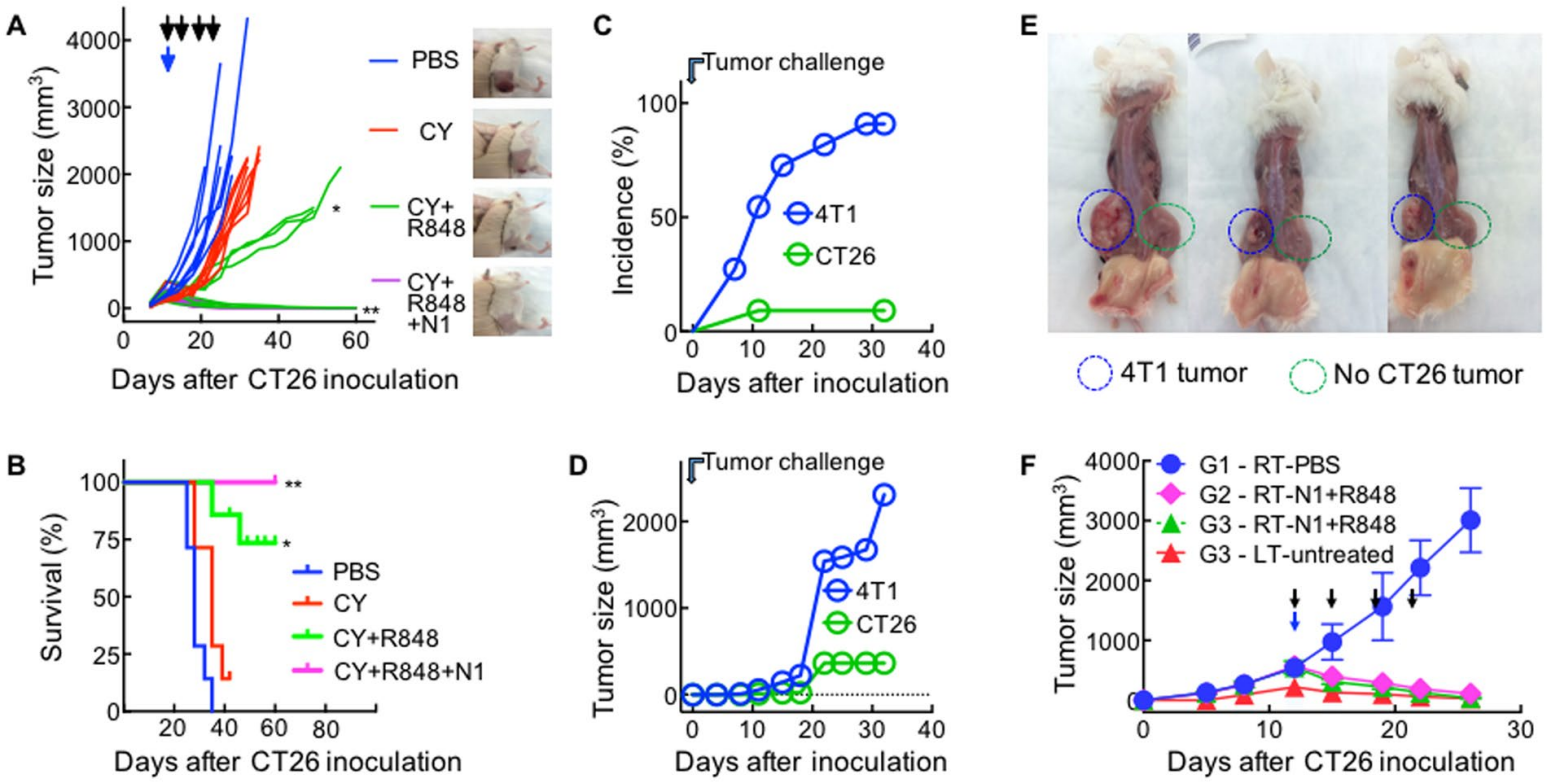
Establishment of a triple combination regimen for the treatment of mice harboring large CT26 colon tumors. (**A** and **B**) Balb/c mice (female, 8 week-old, n = 5) were inoculated s.c. with 2 × 10^5^/mouse of CT26 cells in the right flank on day 1 and the formation of tumors were monitored. When tumors reached approximately 1 cm in diameter (day 12), the mice treated with one dose of i.p. Cytoxan (CY, 2 mg/mouse) on day 12 (blue arrow) and/or four i.t. injections of R848 (10 μg/injection/tumor) or HMGN1 (N1, 10 μg/injection/tumor) on day 12, 15, 19, and 22 (black arrows) in various combinations. Tumor growth (**A** mean ± SD) and survival (**B**) were monitored and plotted (blue circle = PBS, red circle = CY, green circle = CY + R848, purple circle = CY + R848 + N1). The photo image of one mouse of each group on day 21 is also included (**A**). Shown are the results of one experiment representative of three. (**C**–**E**) Ten CT26-free mice resulted from treatment with the combination of CY + R848 + HMGN1 in (**A** and **B** were s.c. inoculated with 2 × 10^5^/mouse of CT26 cells in the right flank and identical amount of 4T1 cells in the left flank. The appearance (**C**) and growth (**D**) of solid tumors were monitored for up to five weeks. The formation of 4T1 tumors but not CT26 tumors were confirmed by photographing of the naked flank regions of three randomly chosen euthanized mice (**E**). (**F**) Three groups (G1-G3) of female Balb/c mice (8 week-old, n = 5) were inoculated s.c. with 2 × 10^5^/mouse of CT26 cells in the right flank on day 1. On day 5, mice in G3 were inoculated s.c. with 2 × 10^5^/mouse of CT26 cells in the left flank. Mice were treated with PBS (G1) or a combination (G2 and G3) of one i.p. Cytoxan (CY, 2 mg/mouse, blue arrow) and four i.t. injections of R848 and HMGN1 (each at 10 μg/injection/tumor, black arrows) into the right tumors twice weekly starting on day 12. Tumor growth (mean ± SD) was monitored and plotted (RT and LT = right and left tumor, respectively). *p < 0.05 and **p < 0.01 when compared with PBS-treated group.

Due to its synergistic effect on DC production of IL-12, a cytokine critical for Th1 polarization, IFNγ and CTL induction^32^, R848 was incorporated into the therapeutic regimen to treat Balb/c mice bearing large (∅ = 0.9~1.1 cm) CT26 tumors. As shown in Fig. 2A, one dose of i.p. CY (2 mg/mouse) only slightly delayed the growth of CT 26 tumors, while the combination of one dose of i.p. CY and four doses of i.t. R848 (10 μg/mouse) slowed the growth of large CT26 tumors. Strikingly, the triple combination of CY, R848 and HMGN1 completely eradicated large established CT26 tumors (Fig. 2A). CT26-bearing mice treated with the triple combination became long-term survivors and were tumor-free (Fig. 2B). When mice cured by the triple treatment were inoculated s.c. with CT26 cells into the right flank and 4T1 (a Balb/c breast cancer cell line) into the contralateral flank, all mice formed 4T1 tumors on the left flank, whereas only one of 10 mice developed a small CT26 tumor on its right flank (Fig. 2C & D) as illustrated (Fig. 2E). Thus, mice cured of the initial large CT26 tumors became specifically resistant to challenge with CT26, but not to unrelated 4T1, tumors, indicative of development of specific antitumor immunity, despite the fact that no exogenous CT26-associated TAAs were administered. This triple therapeutic regimen was dubbed “TheraVac” for short since it cured mice with large tumors and resulted in the generation of tumor-specific immunity against the same tumor.

To simulate metastasis, Balb/c mice were inoculated s.c. with CT26 into the right flank, and 5 days later inoculated with CT26 into the left flank. By day 12, the mice formed large CT26 tumors in the right flank and small tumors in the left flank to imitate primary and metastatic tumors, respectively. CT26-bearing mice were treated by an i.p. dose of CY and 4 i.t. injections of R848 and HMGN1 into the larger right tumors for the subsequent two weeks (Fig. 2F). As anticipated, TheraVac treatment eradicated the large right tumors (G2 & G3 of Fig. 2F). Interestingly, the treatment also caused complete regression of smaller CT26 tumors in the left flank (G3 of Fig. 2F), suggesting that TheraVac was also effective in combating metastatic lesions. Thus, TheraVac treatment induced local as well as systemic antitumor immunity.

**Figure 3:**
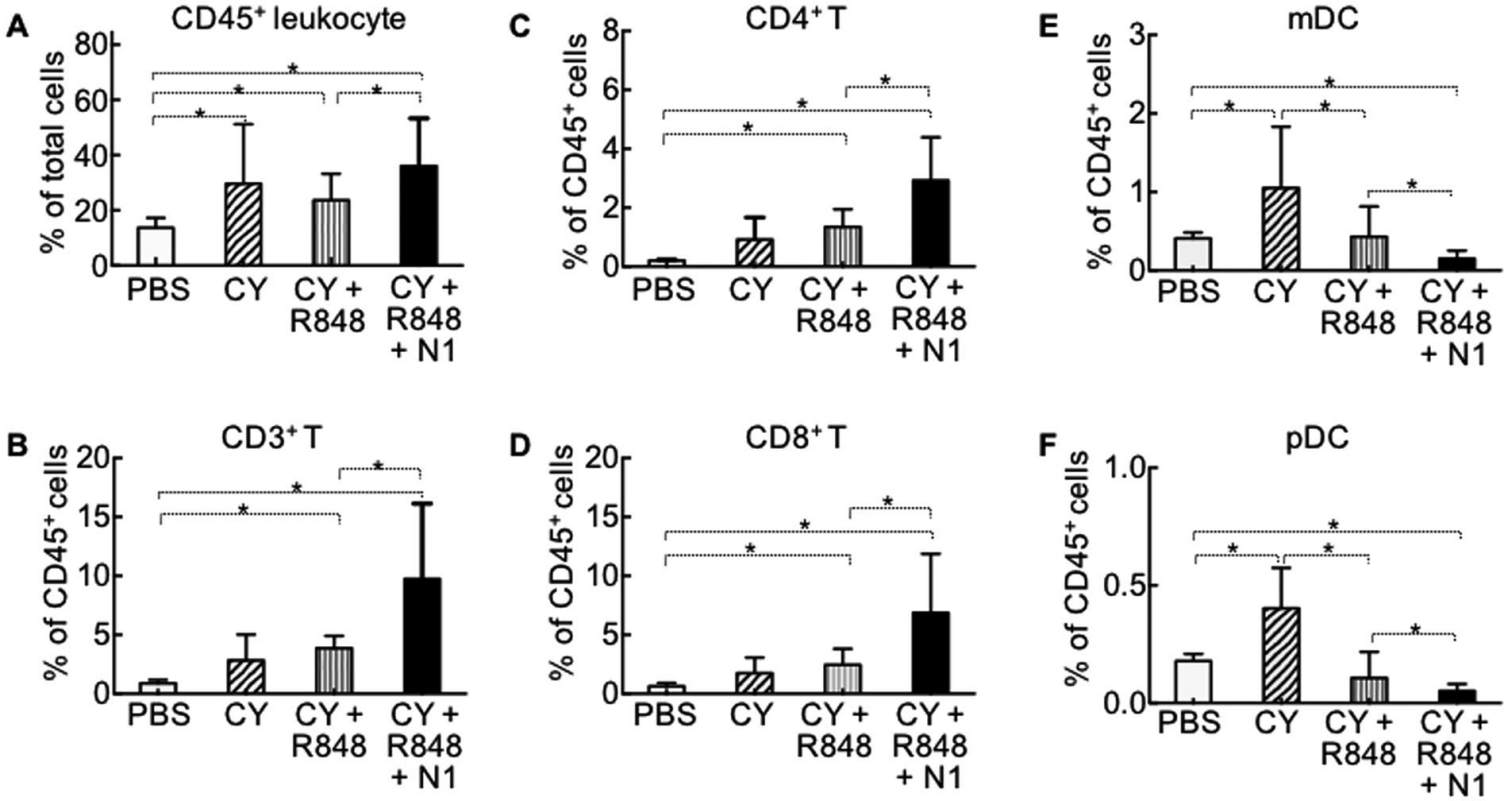
Profiling of tumor-infiltrating leukocytes after treatment with the TheraVac combination of CY, R848, and HMGN1. Four groups of Balb/c mice (female, 8 week-old, n = 5) were inoculated s.c. with 2 × 10^5^/mouse of CT26 cells in the right flank on day 1 and the treatment started on day 12 with one dose of i.p. CY (2 mg/mouse) on day 12 and three i.t. injections of R848 and N1 (both at 10 μg/injection/tumor) on day 12, 15, and 19. Forty-eight hours after the last treatment, the residual tumors were resected *en masse*, and enzymatically digested to make single cell suspension. The single cell suspension was stained with FITC-anti-mouse CD3, PE-anti-mouse CD4, PE-Cy5-anti-mouse-CD8, APC-anti-mouse-CD11c, PerCP-Cy 5.5-anti-mouse CD45, PE-Cy7-anti-mouse-B220, and Pacific Blue-anti-mouse-F4/80, and subsequently analyzed. The average (mean ± SD) of CD45+ leukocytes (**A**), CD3+ T (**B**), CD4^+^ T (**C**), CD8^+^ T(**D**), conventional DC (cDC, (**E**) and plasmacytoid DC (pDC, (**F**) in each group were plotted. cDC and pDC were defined as CD11c^+^/B220^−^ and CD11c^+^/B220^+^, respectively. *p < 0.05. Shown are the results of one experiment representative of three.

To gain insight into the immunologic status of Balb/c mice bearing big CT26 tumors two days after the third TheraVac treatment, residual tumors were resected *en masse*, sliced into approximately 1 mm^3^ pieces, and digested in L15 medium containing a cocktail of collagenase I, collagenase II, collagenase IV, deoxyribonuclease I, and elastase to make single cell suspensions. The cell suspensions were immunostained with various fluorochrome-conjugated antibodies and analyzed by flow cytometry. CY alone moderately increased the infiltration of all leukocytes (Fig. 3A–F). The augmented infiltration by myeloid (CD11c^+^ and B220^−^) and plasmacytoid (CD11c^+^ and B220^+^) tDCs in response to CY (Fig. 3E & F) was likely caused by an increase in the number of peripheral dendritic cells^6,33^. Treatment with the combination of CY and R848 caused moderate increases in the infiltration of CD45^+^ leukocytes (Fig. 3A), CD3^+^ T cells (Fig. 3B), CD4^+^ T (Fig. 3C), and CD8^+^ T cells (Fig. 3D), but the infiltration of myeloid and plasmacytoid tDCs was reduced to the level in the PBS-treated group (Fig. 3E & F). The TheraVac combination of CY, R848 and HMGN1 markedly increased the infiltration of CD45^+^ leukocytes (Fig. 3A), CD3^+^ T cells (Fig. 3B), CD4^+^ T (Fig. 3C), and CD8^+^ T cells (Fig. 3D), and markedly lowered the percentage of myeloid and plasmacytoid tDCs (Fig. 3E & F). Therefore, the curative effect of the TheraVac on large CT26 tumors was accompanied by augmented infiltration of effector T cells into the tumor tissue.

**Figure 4:**
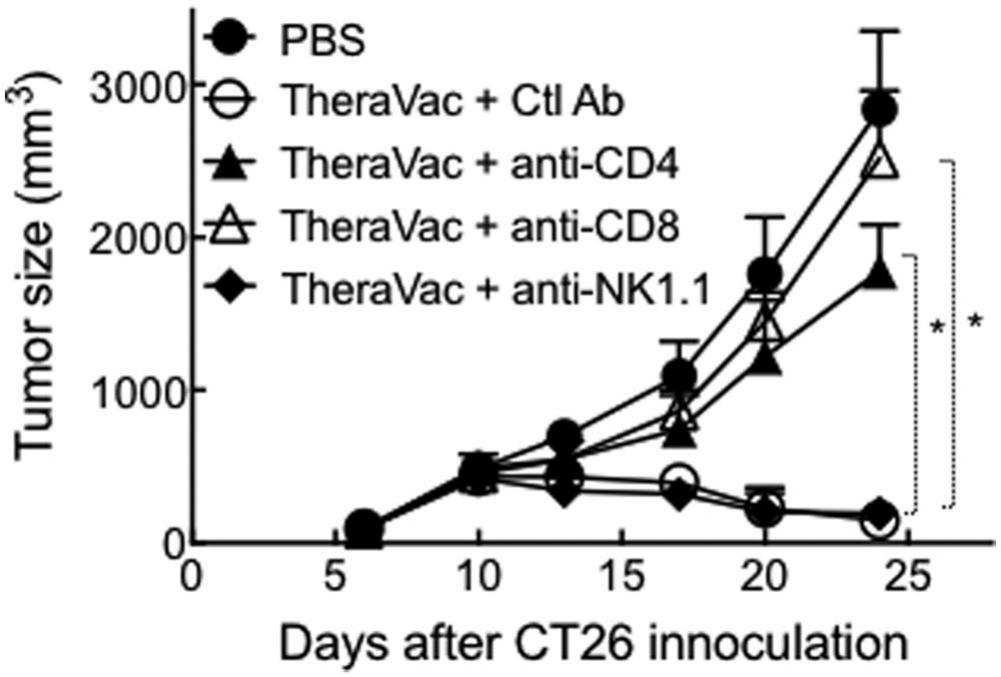
Simultaneous administration of anti-CD4 or anti-CD8 antibody inhibited the therapeutic efficacy of TheraVac consisting of CY, R848, and HMGN1. Five groups of Balb/c mice (female, 8 week-old, n = 5) were inoculated s.c. with 2 × 10^5^/mouse of CT26 cells in the right flank on day 1 and the treatment started on day 10 with one dose of i.p. CY (2 mg/mouse) on day 10 and four i.t. injections of R848 and N1 (both at 10 μg/injection/tumor) on day 10, 13, 17, and 20. Antibodies including control antibody (Ctl Ab), anti-CD4, anti-CD8, or anti-NK1.1 were administered i.p. at 200 μg/injection/mouse on day 10, 13, 17, and 20. The mice were monitored for tumor growth. *p < 0.001 when compared with TheraVac + Ctl Ab group. Similar results were obtained from two independent experiments.

To investigate whether the therapeutic antitumor effect of TheraVac consisting of CY, R848, and HMGN1 was due to generation of antitumor immunity, neutralizing anti-CD4, anti-CD8, or anti-NK1.1 antibody was used together with TheraVac. As shown in Fig. 4, CT26 tumor growth was halted by TheraVac. Simultaneous administration of anti-CD4 or anti-CD8 antibody almost completely nullified the therapeutic effect of TheraVac, demonstrating that both CD4 and CD8 T cells are required for the manifestation of the antitumor effect of TheraVac. In contrast, simultaneous administration of anti-NK1.1 antibody did not affect the therapeutic antitumor effect of TheraVac, suggesting that NK cells did not contribute significantly.

**Figure 5:**
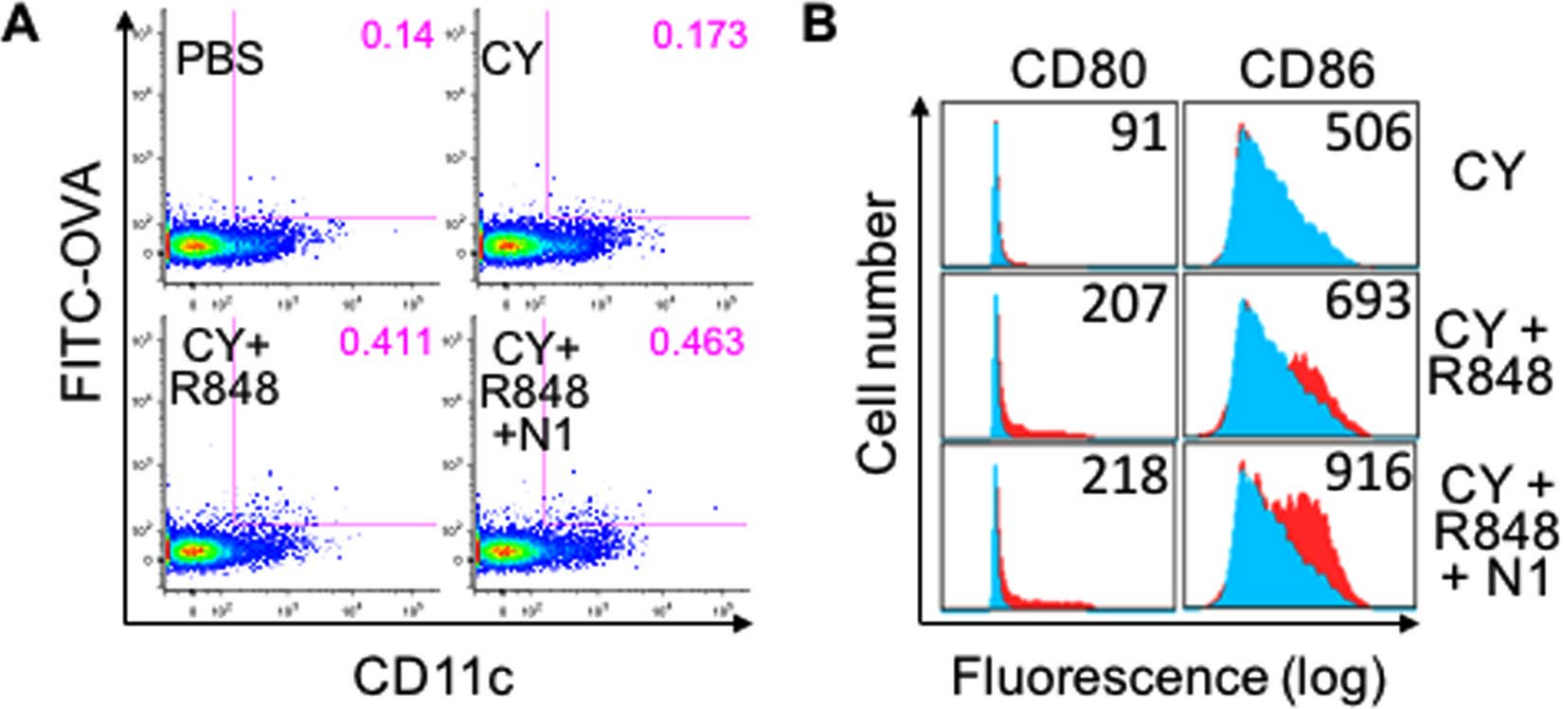
Enhancement of tiDC activation and homing to draining lymph nodes upon treatment with TheraVac combination of CY, R848, and HMGN1. Balb/c mice (female, 8 week-old, n = 3) were inoculated s.c. with 2 × 10^5^/mouse of CT26 cells in the right flank on day 1. On day 12, CT26-bearing mice were i.t. injected with 50 μl of PBS containing 1 μg FITC-conjugated OVA and treated on day 13 with i.p. CY (2 mg/mouse), i.t. R848 and N1(both at 10 μg/tumor). The draining inguinal lymph nodes were removed 24 h later to make single cell suspensions, which were stained with PE-anti-mouse CD80, PE-Cy5-anti-mouse CD86, and APC-anti-mouse CD11c. Dot plot of one representative mouse of each group is shown, with the % of tDCs in the draining lymph node highlighted in pink (**A**). Surface CD80 and CD86 expression were shown by overlay histogram with control group in light blue and treated groups in red. The numbers inside the histograms are the average geometric mean of CD80 or CD86 of the same group.

Given the DC-activating effects of R848 and HMGN1^6,27^ as shown by data in Fig. 1, we hypothesized that tDCs might mature and migrate to the draining lymph nodes (LN) after TheraVac therapy. To examine this possibility, Balb/c mice harboring large CT26 tumors were injected i.t. with FITC-labeled ovalbumin and subsequently treated with PBS, CY, CY + R848, or the TheraVac combination. Twenty-four hours after treatment, draining LNs were harvested, sliced, and enzymatically digested to make single cell suspensions. The cell suspensions were immunostained with various fluorochrome-conjugated antibodies against mouse CD11c, CD80, and CD86, and analyzed on a BD LSR II flow cytometer. Gating on DCs (CD11c^+^) that were FITC^+^ showed that CT26-bearing mice treated with TheraVac had the highest number of FITC^+^ DCs in the draining inguinal LNs, followed by mice treated with CY + R848 (Fig. 5A), demonstrating that TheraVac treatment resulted in homing of tDCs into the draining LN. Mice treated with CY alone did not significantly promote tDC homing to the draining inguinal LN (Fig. 5A). Comparison of the levels of costimulatory molecule expression by CD11^+^ DCs revealed that DCs in the draining LNs of CT26-bearing mice treated with TheraVac exhibited the highest levels of CD80 and CD86 (Fig. 5B). DCs in the draining LN of CT26-bearing mice treated with CY + R848 also had higher expression of CD80 and CD86, whereas treatment with CY alone did not upregulate DC expression of CD80 and CD86 (Fig. 5B). We propose that the TheraVac regimen promoted activation and homing of tDCs to LN for the induction of specific immunity against CT26, which led to the eradication of CT26 tumors as well as resistance to re-challenge with CT26 tumor inoculation.

**Figure 6:**
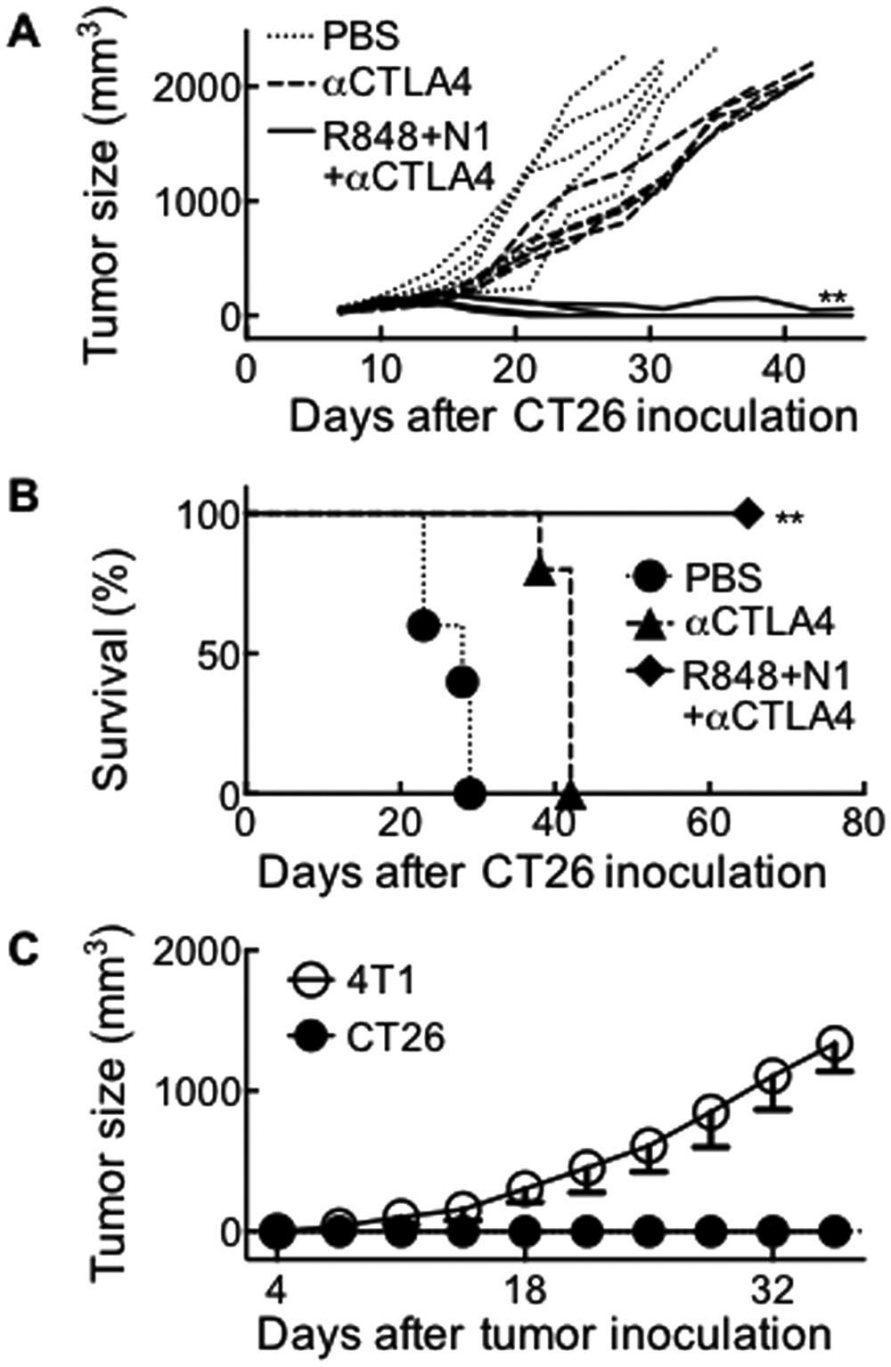
Eradication of large CT26 tumors by TheraVac consisting of HMGN1, R848 and anti-CTLA4. Versatility of TheraVac regimen. Balb/c mice (female, 8 week-old, n = 5) were inoculated s.c. with 2 × 10^5^/mouse of CT26 cells in one flank on day 1. After tumors reached 0.9–1.2 cm in diameter, mice were i.t. injected with 0.1 ml PBS or PBS containing R848, HMGN1 (N1) and anti-CTLA4 (10 μg each) on day 12, 15, 19, and 21. The mice were monitored for tumor growth (**A**) and survival (**B**). Eight weeks after the treated mice became tumor-free, they were s.c. inoculated with identical number (2 × 10^5^/mouse) of CT26 and 4T1 cells in contralateral flanks and monitored for the formation and growth of CT26 and 4T1 tumors (**C**). **p < 0.01 when compared with PBS-treated group.

Next, we investigated whether CY in the TheraVac regimen could be replaced by other immune checkpoint inhibitor(s). Balb/c mice bearing large (∅ = 0.9~1.1 cm) CT26 tumors were treated twice weekly with either PBS as a negative control or a combination of HMGN1 (10 μg/mouse), R848 (10 μg/mouse), and anti-mouse CTLA4 (10 μg/mouse). Four rounds of treatment with R848, HMGN1, and anti-CTLA4 eradicated large CT26 tumors (Fig. 6A). The treated mice became tumor-free and survived for up to two months (Fig. 6B). Upon challenge of those tumor-free mice with CT26 and 4T1 tumor cells on their contralateral flanks, all the mice formed continuously growing 4T1 solid tumors, while none of the mice formed CT26 tumors, demonstrating the acquisition of tumor-specific immunity (Fig. 6C).

**Figure 7:**
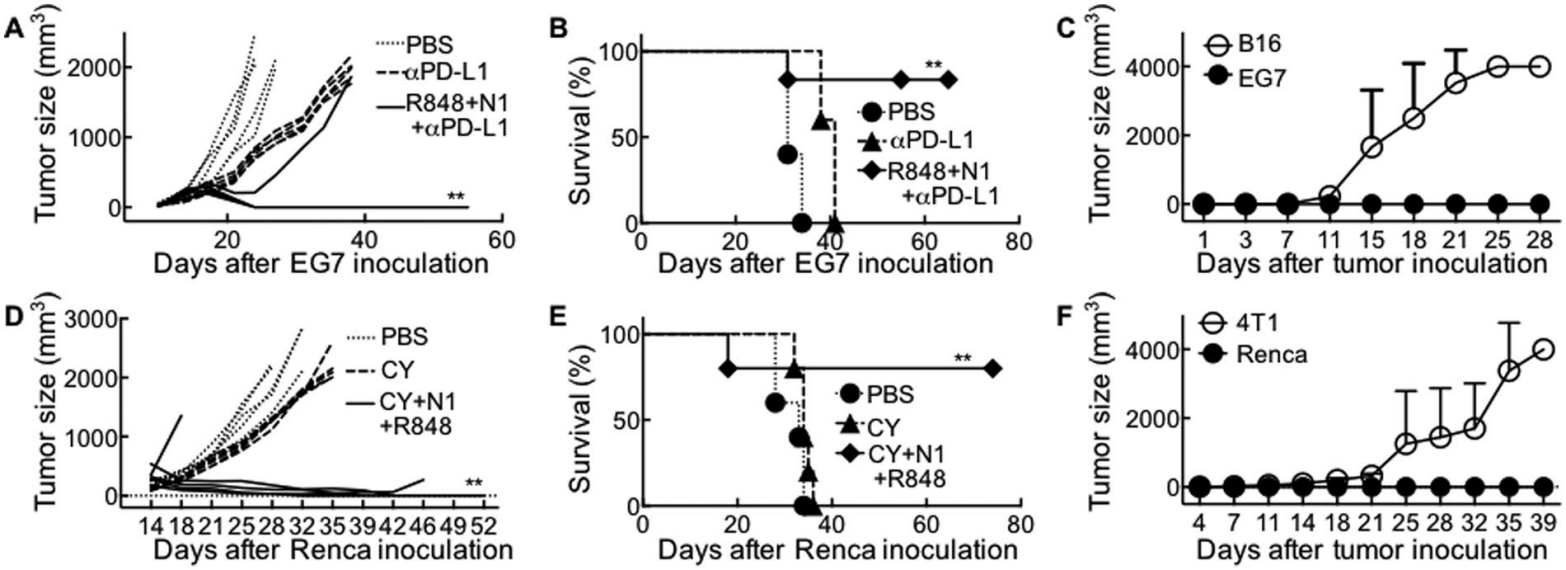
Treatment of large mouse EG7 and Renca tumors by TheraVac. (**A**–**C**) C57BL/6 mice (female, 8 week-old, n = 5) were inoculated s.c. with 0.2 ml PBS containing 2 × 10^5^ EG7 mouse thymoma cells in one flank on day 1. After tumors reached approximately 1 cm in diameter on day 14, mice were i.t. injected with 0.1 ml PBS or PBS containing R848, HMGN1 (N1) and anti-PD-L1 (10 μg each) on day 14, 17, 21, and 24. The mice were monitored for tumor growth (**A**) and survival (**B**). Eight weeks after the treated mice became tumor-free (4/5), the four tumor-free were s.c. inoculated with identical number (2 × 10^5^/mouse) of EG7 and B16 (F10) melanoma cells in contralateral flanks and monitored for the formation and growth of CT26 and 4T1 tumors (**C**). All data are representative of at least two experiments. (**D**–**F)** Balb/c mice (female, 8 week-old, n = 5) were inoculated s.c. with 1 × 10^6^/mouse of Renca renal carcinoma cells in one flank. After tumors reached approximately 1 cm in diameter, mice were treated with one i.p. injection of CY (2 mg/mouse) on day 13, and four i.t. injected with 50 μl PBS containing 10 μg of R848 and HMGN1 (N1) on day 13, 18, 22, and 25, with identical amount of PBS injected into control Renca-bearing mice. The mice were monitored for tumor growth (**D**) and survival (**E**). Four of five treated mice became tumor-free, which, after eight weeks, were s.c. inoculated with 2 × 10^5^/mouse of 4T1 cells and 1 × 10^6^/mouse of Renca cells in contralateral flanks. The formation and growth of 4T1 and Renca were monitored and plotted (**F**). All data are representative of at least two experiments. *p < 0.05 and **p < 0.01 when compared with PBS-treated group.

Lastly, we determined whether TheraVac consisting of R848, HMGN1, and a checkpoint inhibitor (e.g. CY, anti-CTLA4, or anti-PD-L1) could be used to treat several other types of large solid tumors in different mouse strains. C57BL/6 mice bearing large EG7 tumors were successfully treated with four rounds of i.t. injection of R848 (10 μg/mouse/injection), HMGN1 (10 μg/mouse/injection), and anti-PD-L1 (10 μg/mouse/injection) within two weeks, with 4 of the 5 mice becoming tumor-free (Fig. 7A & B). The four cured mice were resistant to re-challenge with EG7, but not resistant to inoculation with unrelated B16 melanoma (Fig. 7C). Therefore, CY can be replaced by checkpoint inhibitors in the TheraVac regimen to treat C57BL/6 mice bearing large established EG7 tumors. When 5 Balb/c mice bearing large Renca tumors were treated with i.p. injection CY and i.t. injection of R848 + HMGN1, four were cured (Fig. 7D) and became tumor-free for more than 2 months (Fig. 7E). Inoculation of these cured mice with 4T1 and Renca tumors cells on the contralateral flanks resulted in the formation of 4T1, but not Renca, tumors, demonstrating that TheraVac treatment conferred Renca-specific immunoprotection (Fig. 7F). Additionally, C57BL/6 mice harboring large EG7 tumors were successfully treated with TheraVac consisting of anti-CTLA4, R848, and HMGN1 (sFig. 3). Thus, TheraVac appears to be effective against multiple tumor types.

## Discussion

We have developed a TheraVac regimen consisting of two arms: one arm consisting of i.t. administration of R848 and HMGN1 which promoted the activation and homing of tDCs; the other arm consisting of a inhibitor (e.g. low dose of CY, anti-CTLA4 or anti-PD-L1) which countered the immunosuppression in the tumor tissues and tumor-bearing hosts. This dual TheraVac approach in the absence of TAA administration not only caused complete regression of large solid tumors, but also resulted in the acquisition of specific protective immunity against the treated tumor, indicative of successful vaccination. The capacity of TheraVac to act as an antitumor vaccine is based on the activation of tDCs by the administered combination of R848 and HMGN1, which promotes tDC homing to draining lymph nodes for the induction of specific protective anti-tumor immune responses. This notion is also supported by the fact that the therapeutic effect of TheraVac was dependent on the presence of both CD4 and CD8 T cells (Fig. 4). Normally, tDCs in large experimental tumors or late-stage human tumors, although loaded with TAAs, are mostly immature and exhibit a paralyzed phenotype that lack the capacity to induce the generation of antitumor immune responses^6,8,10,11,14^. It is therefore essential to induce robust activation of tDCs in order to promote their homing to draining LNs for the induction of antitumor immune responses. Intratumoral delivery of HMGN1 and R848 in the TheraVac regimen was sufficient to activate tDCs as evidenced by the greatly reduced numbers of both myeloid and plasmacytoid tDCs in TheraVac-treated tumors (Fig. 3E & F) as well as augmented activation and homing to draining LNs (Fig. 5).

Simultaneous use of HMGN1 and R848 was important for the effective eradication of large tumors by TheraVac (Figs 2, 6, 7, and sFig. 3) since either HMGN1 + CY (data not shown) or R848 + CY (Fig. 2) was not sufficient to eradicate large established CT26 tumors. There are two major reasons that account for the greater effectiveness of combinational intratumoral delivery of HMGN1 and R848. First, HMGN1 and R848 activate DCs through TLR4 and TLR7/8, respectively, to preferentially induce Th1 and CTL responses^27,34^. Although the signal transduction pathways of TLR4 and TLR7/8 differ, the both culminate in the activation of NF-κB and IRF7, two transcriptional activators of DC maturation/activation. Therefore, simultaneous treatment with HMGN1 and R848 can more robustly activate tDCs (Fig. 5). This was also attested by data showing that HMGN1 and R848 synergistically upregulated DC production of IL-12 (Fig. 1), an excellent inducer of Th1 immune response and IFNγ production. Secondly, tDCs are heterologous, consisting of at least mDCs and pDCs^14^. In both humans and mice, TLR4 is expressed by mDCs whereas functional TLR7/8 is specifically expressed by pDCs^14,34,35^. The combination of HMGN1 and R848 would lead the activation of both myeloid and plasmacytoid tDCs, as evidenced by their reduction in the treated tumors (Fig. 3). Thus, simultaneous administration of HMGN1 and R848 into tumor tissues activates tDCs and promotes potent protective anti-tumor immunity (Figs 2–7).

Several TLR agonists capable of promoting Th1 immune responses have been tested for the treatment of cancers. CpG ODN has antitumor activity in many mouse models, however, it does not work well in humans perhaps due to the fact that TLR9 is expressed by mouse macrophages, mDCs, and pDCs, but only by human pDCs^36–38^. PolyI:C, an agonist for TLR3 and intracellular RNA sensors such as MDA5, RIG-I, DDX1, etc., is a potent DC activator and has been tested in multiple tumor models^39^, but it causes intolerable endotoxin-like cytokine toxicity in humans^40^. TLR4 is expressed by mDCs and it’s activation is particularly important for the induction of IL-12 and development of Th1 adaptive immune responses^32,41^. However, LPS, the natural ligand of TLR4, is not applicable for cancer immunotherapy due to severe systemic toxicity. TLR4 ligands with less toxicity (e.g. monophosphoryl lipid A or synthetic lipid A analogue) have therefore been identified and tested^42,43^. Unfortunately, clinical trials with more than 300,000 human cancer patients treated with various vaccines containing monophosphoryl lipid A alone or combined with other immunostimulants such as saponin QS-21 (as in AS01 and AS02) have demonstrated only very limited clinical success^44^. TheraVac provides a novel tumor immunotherapeutic regimen that has great potential to be translated into humans because 1) the synergistic DC-activating effect of HMGN1 and R848 was seen both in mouse and human DCs (Fig. 1); 2) the inhibitors of immunosuppression in TheraVac, either CY, anti-PD-L1, or anti-CTLA4, has already been shown to be beneficial for human cancer patients^1,2,5,45^; and 3) TheraVac was effective on large established mouse tumors that more closely simulate human cancers.

It is noteworthy that large tumors of different types regressed after four TheraVac treatment within two weeks (Figs 2, 5 & 6), attesting to the effectiveness of the TheraVac regimen. No obvious side effect was observed for TheraVac. Mice harboring large tumors treated with TheraVac did not experience significant loss of body weight or patchy hair loss (data not shown), indicative of undesirable side effects such as autoimmune responses. However, tumor-bearing mice treated with a combination of HMGN1, R848, and systemic administration of both anti-CTLA4 and anti-PD-L1 together manifested significant loss of body weight (>20%) and patchy hair/hair color (data not shown), suggesting that simultaneous usage of anti-CTLA4 and anti-PD-L1 poses increased risk of unleashing autoimmune response(s).

It has not previously been shown that promoting the endogenous antitumor immune responses can consistently eradicate large established mouse tumors in immunocompetent mice. A very recent publication reported a complex combinational immunotherapic regimen consisting of four agents including a TAA-targeting antibody, IL-2, anti-PD-L1, and a potent T cell vaccine made of TAA peptide and CpG ODN, which was shown to be effective against large mouse B6 melanoma, DD-Her2/neu breast carcinoma, and TC-1 lung tumor^46^. In comparison, TheraVac offers unique advantages. TheraVac was effective against multiple types of tumors in two mouse strains (Figs 2, 5, 6, and sFig. 3) and can therefore be potentially used to treat diverse types of accessible human cancers such as head-and-neck carcinoma, breast cancer, skin melanoma. Additionally, TheraVac achieves successful tumor vaccination without relying on the use of exogenous TAAs, bypassing the necessity for the identification of TAAs. Furthermore, TheraVac eradicates large established tumors without the use of high dose of chemotherapeutic agents or radiation and hence should have lower side effects in clinical trials. Lastly, TheraVac does not required a TAA-specific antibody, and therefore can potentially be used for the treatment of all cancers. The only drawback is the need to inject R848 and HMGN1 into the tumor tissues, which may not be easily accessible for tumors in deep organs. We are investigating means of overcoming this limitation by using nanoparticle platforms for the delivery of R848 and HMGN1 since many nanoparticle delivery systems can deliver payload into tumor tissues upon systemic administration^47^.

## Methods

### Mice and cell lines

Balb/c and C57BL/6 mice (8–12-week old, female) were provided by the Animal Production Area of the NCI (Frederick, MD). NCI-Frederick is accredited by AAALAC International and follows the Public Health Service Policy for the Care and Use of Laboratory Animals. Animal care was provided in accordance with the procedures outlined in the “Guide for Care and Use of Laboratory Press” (Washington, D.C.). All animal studies were approved by the Institutional Animal Care and Use Committee (IACUC) of National Cancer Institute at Frederick (Frederick, MD).

All cell lines [CT26 colon cancer cell line (CRL-2638), 4T1 breast cancer cell line (CRL-2539), Renca renal ademocarcinoma cell line (CRL-2947), EG7 thymoma cell line (CRL-2113), and B16F10 melanoma cell line (CLR-6475) used in the present study were initially purchased from the American Type Culture Collection (ATCC, Manassas, VA). CT26, 4T1, and EG7 were maintained in the laboratory, while Renca was obtained from Dr. Jonathan Weiss of the Cancer and Inflammation Program, NCI at Frederick. All cell lines were examined for contaminants with Molecular Testing of Biological Materials (MTBM) test (Animal Health Diagnostic Laboratory, NCI-Frederick). The morphology, *in vitro* and *in vivo* growth rate and metastatic ability of cell lines were routinely monitored for stability and consistency. All cell lines were maintained in RPMI-1640 medium [RPMI-1640 (Meditech) supplemented with 10% FBS (Hyclone) and 2 mmol/L glutamine, 25 mmol/L HEPES, 100 U/ml penicillin, 100 ug/ml streptomycin, and 50 umol/L 2-mercaptoethanol] at 37 °C in a humidified incubator with 5% CO_2_.

### Generation and treatment of DCs

Mouse bone marrow (BM) -derived DCs were generated as previously reported^48^. Briefly, BM progenitors isolated from the femurs and tibias were incubated at 5 × 10^5^ cells/ml in generating medium (RPMI 1640 containing 10% FBS [Hyclone], 2 mmol/L glutamine, 25 mmol/L HEPES, 100 U/ml penicillin, 100 μg/ml streptomycin, and 50 μmol/L 2-mercaptoethanol and 20 ng/ml of mouse GM-CSF (PeproTech) at 37 °C in a humidified incubator with 5% CO_2_ for 6 d to generate immature DCs. Subsequently, immature DCs were incubated in fresh culture medium at 5 × 10^5^ cells/ml in the absence (sham) or presence of HMGN1, R848 (InvivoGen), or LPS (Sigma-Aldrich, as positive control) alone or in combination at specified concentrations for 6–48 h at 37 °C in a humidified incubator with 5% CO_2_ before analyzing their function and phenotype. Recombinant HMGN1 used in the present study was generated in insect cells using a baculovirus expressing system and purified as previously described^27^.

For the generation of human DCs, peripheral blood monocytes were isolated and cultured in the presence of 50 ng/ml of human GM-CSF (PeproTech) and IL-4 (PeproTech) for 5 to 7 days as previously described^48^.

### Quantitative real-time polymerase chain reaction (qRT-PCR)

Total RNA was extracted from human or mouse DCs after 6–8 hours of stimulation with HMGN1 or R848 using an RNeasy Micro Kit (Qiagen, Hilden, Germany). cDNA was synthesized from RNA with QuantiTect Reverse Transcription Kit (Qiagen). For qRT-PCR, TaqMan Universal PCR Master Mix and TaqMan Gene Expression Assays (Applied Biosystems, Foster City, CA) were applied to measure the expression of mouse or human cytokines using specific primers obtained from Qiagen (sTable 1). qRT-PCR amplifications were conducted in a thermocycler DNA Engine (OPTICON2; Bio-Rad Laboratories, Hercules, CA). The cycling conditions for PCR amplification were 50 °C for 2 minutes and 95 °C for 10 minutes, followed by 45 cycles of 95 °C for 15 seconds and 60 °C for 1 minute. The expression levels of IL-12, TNF-α and β-actin in mouse or human DC were analyzed by qRT-PCR (Applied Biosystems).

### Mouse tumor models and treatment

Female mice (Balb/c or C57BL/6, n = 5–10, 8–12-wk old) were subcutaneously injected with 0.1 ml PBS containing CT26 (2 × 10^6^/ml), 4T1 (2 × 10^6^/ml), Renca (1 × 10^7^/ml), EG7 (2 × 10^6^/ml), or B16F10 (2 × 10^6^/ml) into flank (left or right) regions as previously reported^19,49,50^. The appearance and size of tumors as well as the mouse body weight were monitored twice weekly. The length (L) and width of tumors were measured with a caliper. Tumor size was calculated by the formula: (L X W^2^)/2. When tumors reached a small (5–7 mm) or big size (9–11 mm in diameter), the tumor-bearing mice were treated with intraperitoneal (i.p.) injection of Cytoxan (2 mg/0.2 ml/mouse), intratumoral (i.t.) injection of anti-CTLA4 (10 μg/0.05 ml/mouse), anti- PD-L1 (10 μg/0.05 ml/mouse), i.t. injection of HMGN1 (10 μg/0.05 ml/mouse), and i.t. injection of R848 (10 μg/0.05 ml/mouse) alone or in combination as specified using PBS as negative control. Anti-CTLA4 (clone UC10–4F10-11), anti-PD-L1 (clone 10 F.9G2), and respective control Armenian Hamster IgG or rat IgG2b (clone LTF-2) were purchased from Bio X Cell (West Labanon, NH). For depleting CD4, CD8, or NK cells, mice were injected intraperitoneally with 0.2 ml PBS containing 200 μg of either control rat IgG (clone 2A3, BioXcell, West Lebanon, NH), anti-mouse CD4 (clone GK1.5, BioXcell), anti-mouse CD8α (clone 53-6.72), or anti-mouse NK1.1 (clone PK136, BioXcell) simultaneously with TheraVac treatment. Tumor-free mice were re-challenged with the same or unrelated tumors on contralateral flanks and the formation and growth of tumor were monitored.

To monitor tDC migration to draining lymph nodes, mice harboring big tumors were i.t. injected with FITC-OVA (Molecular Probe) at 1 μg/50 μl PBS/tumor, and treated with i.p. injection of CY (2 mg/0.2 ml PBS/mouse) or i.t. injection of R848 (10 μg/50 μl PBS/tumor) and HMGN1 (10 μg/50 μl PBS/tumor). Twenty-four hours after the treatment, the draining inguinal lymph nodes were removed for preparing single cell suspensions.

### Dissociation of tumors and draining lymph nodes

Tumors and draining LNs were resected, sliced into approximately 1 mm^3^ cubes, and digested at 25 ml/gram for two rounds in a enzymatic cocktail at 37 °C for 45 minutes with constant slow shaking (~80 rpm), with vigorous pipetting in-between. The enzymatic cocktail was Leibovitz L-15 medium (Meditech) containing 0.17 mg/ml collagenase I (Worthington Biochemical Corp., Lakewood, NJ), 0.056 mg/ml collagenase II (Worthington Biochemical), 0.17 mg/ml collagenase VI (Worthington Biochemical), 0.025 mg/ml deoxyribonuclease I (Worthington Biochemical), and 0.025 mg/ml elastase (Worthington Biochemical) as previously reported^51^. The final mixtures were passed through 70-um cell strainers, washed three times with PBS, and suspended in PBS to make a single-cell suspension.

### Immunostaining and flow cytometry

For the detection of surface markers, mouse DCs were washed in FACS buffer (PBS containing 0.5% BSA 0.05% NaN_3_), blocked with FACS buffer containing 2% normal mouse serum on ice for 10 minutes, and stained with a combination of FITC-anti-mouse I-A/E (BD/Pharmingen, clone 2G9), PE-anti-mouse CD80 (BD/Pharmingen, clone 16-10A1), PerCP-Cy5-anti-mouse CD86 (BD/Pharmingen, clone GL1), and APC-anti-mouse CD11c (BD/Pharmingen, clone HL3) on ice for 30 minutes. After washing for 3 times with FACS buffer, data from the stained samples were acquired using a BD LSRII multichannel cytometer (Mountain View, CA). For detecting the infiltration of various subsets of leukocytes in the tumor tissues, single cell suspensions of digested tumor tissues were stained with FITC-anti-mouse CD3 (Tonbo Bioscience, clone 145-2C11), PE-anti-mouse CD4 (Maker, clone RM4-5), PE-Cy5-anti-mouse-CD8 (Maker, clone 53-6.7), APC-anti-mouse-CD11c (BD/Pharmingen), PerCP-Cy 5.5-anti-mouse CD45 (BD/Pharmingen, clone 30-F11), PE-Cy7-anti-mouse-B220 (BD/Pharmingen, clone RA3-6B2), and Pacific Blue-anti-mouse-F4/80 (eBioscience, clone RM8) and the stained samples were analyzed and collected on a BDFortessa multichannel cytometer (Mountain View, CA). To determine the maturation and homing of tDCs to draining LNs, single cell suspensions of draining LNs were stained with a combination of PE-anti-mouse CD80 (BD/Pharmingen), PerCP-Cy5-anti-mouse CD86 (BD/Pharmingen), and APC-anti-mouse CD11c (BD/Pharmingen), and data were acquired using a BD LSRII cytometer. All data analyses were performed using the software FlowJo (Tree Star Inc., Ashland, OR).

### Statistics

Unless otherwise specified, all experiments were performed at least three times, and the results of one representative experiment or the mean of multiple experiments are shown. Differences in the *in vivo* tumor growth were determined by repeated measures of ANOVA, whereas differences between other control groups and experimentally treated groups were evaluated by one-way ANOVA after arcsine square-root transformation. The percent of survival data were compared and analyzed by Logrank test, using Graphpad Prism 6.0.

## Electronic supplementary material

Supplemental figures and information
