# Supplementary material for: Development of a Curative Therapeutic Vaccine (TheraVac) for the Treatment of Large Established Tumors
Source: Sci Rep. 2017 Oct 27;7:14186. doi: 10.1038/s41598-017-14655-8 (PMC5660153; doi:10.1038/s41598-017-14655-8)
Supplement: Supplementary file 1 — Supplemental figures and information [file 41598_2017_14655_MOESM1_ESM.pdf]

Development of a Curative Therapeutic Vaccine (TheraVac) for the Treatment of Large Established Tumors

Yingjie Nie[1,2], De Yang[1*], Anna Trivett[1], Zhen Han[1], Haiyun Xin[1], Xin Chen[3,1], Joost J. Oppenheim[1*]

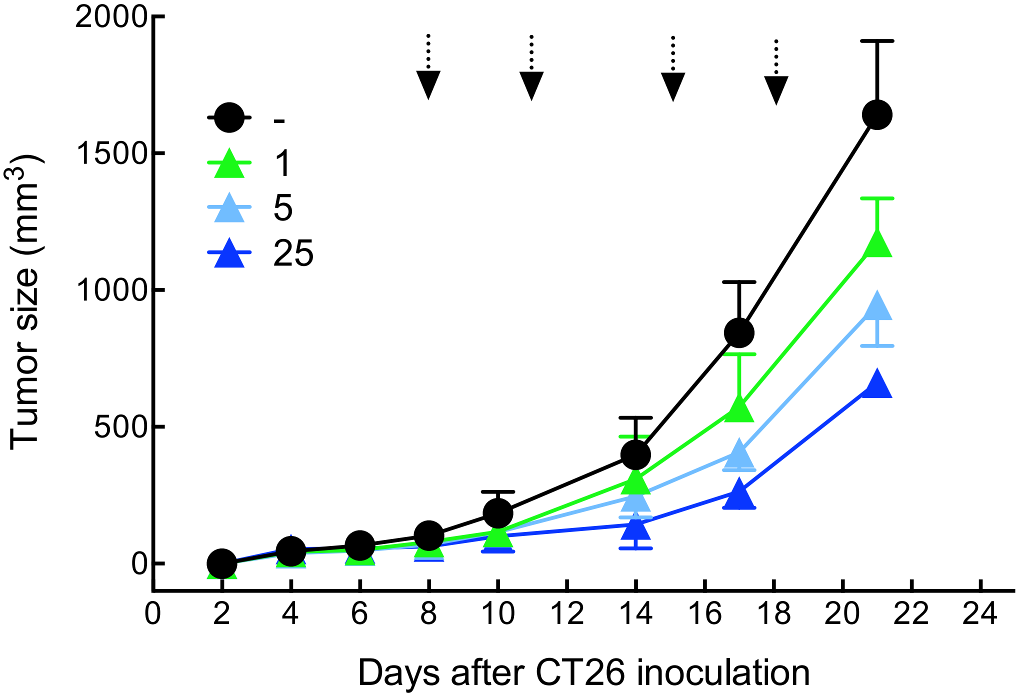

sFig. 1. HMGN1 inhibited the growth of CT26 tumors in a dose-dependent manner. Female Balb/c mice (n = 5, 8-wk old) were subcutaneously injected with 0.1 ml PBS containing CT26 ($2x10^6$/ml) on day 1 and treated with intratumoral injection of 50 ml PBS containing 0, 1, 5, or 25 mg HMGN1 on day 8, 11, 15, and 18 (arrow). Tumor growth was monitored by measuring the length (L) and width (W) of the tumors and calculated by the formula: $(L X W^2)/2$.

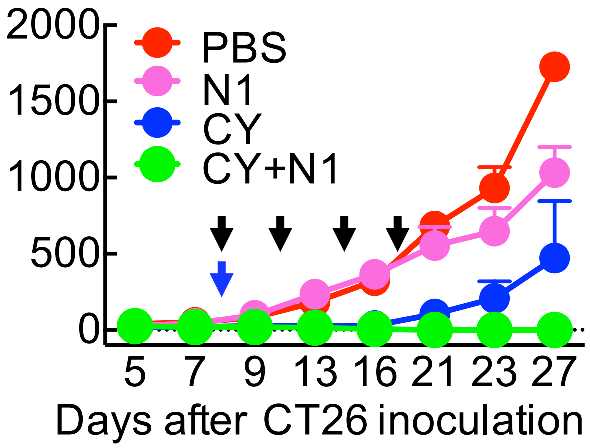

sFig. 2. Combination of HMGN1 and cytoxan eradicated solid CT26 tumors. Balb/c mice (female, 8 week-old, n = 5) inoculated s.c. with $2x10^5$/mouse of CT26 cells were treated with one dose of i.p. Cytoxan (CY, 2 mg/mouse) on day 8 (blue arrow) and four i.t. injections of HMGN1 (N1, 10 μg/injection/tumor) on day 8, 11, 15, and 18 (black arrows) alone or in combination. Tumor formation and growth were monitored as indicated and tumor size was plotted. Tumor growth was monitored by measuring the length (L) and width (W) of the tumors and calculated by the formula: $(L \times W^2)/2$.

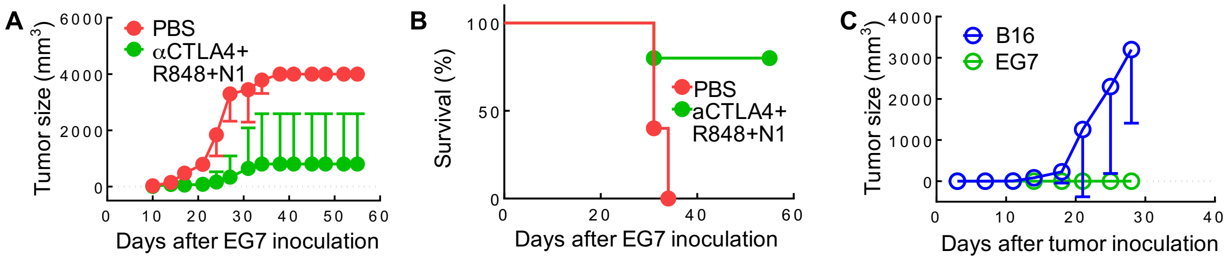

sFig. 3. C57BL/6 mice (female, 8 week-old, n = 5) were inoculated s.c. with 0.1 ml PBS containing 2x10^5 EG7 mouse thymoma cells in one flank on day 1. After tumors reached approximately 1 cm in diameter, mice were treated by i.t. injection 0.1 ml PBS containing R848, HMGN1 (N1) and anti-CTLA4 (10 μg each) on day 14, 17, 21, and 24, with identical amount of PBS injected into control EG7-bearing mice. The mice were monitored for tumor growth (**A**) and survival (**B**). Four of five treated mice became tumor-free, which, after eight weeks, were s.c. inoculated with identical number (2x10^5/mouse) of EG7 and B16 (F10) cells in contralateral flanks. The formation and growth of B16 and EG7 were monitored and plotted (**C**).

sTable 1. Qiagen primers used for measurement of human and mouse cytokines

Target gene	Cat #	Ref position
Human IL-6	PPH00560C-200	795 (NM_000600)
Human TNFα	PPH00341F-200	794 (NM_000594)
Human IL-10	PPH00572C-200	148 (NM_000572)
Human IL-12p40	PPH00545A-200	229 (NM_002187)
Human β actin	PPH00073G-200	730 (NM_001101)
Mouse IL-6	PPM03015A-200	73 (NM_031168)
Mouse TNFα	PPM03113G-200	1940 (NM_013693)
Mouse IL-10	PPM03017C-200	104 (NM_010548)
Mouse IL-12p40	PPM03020E-200	1110 (NM_008352)
Mouse β actin	PPM02945B-200	533 (NM_007393)
